# Structured Multidisciplinary Care for Clinically Complex Kidney Transplant Recipients: A 12‐Month Single‐Center Cohort on Multidimensional Outcomes

**DOI:** 10.1155/joot/4688022

**Published:** 2026-06-17

**Authors:** Beatriz Rodríguez Cubillo, Maria Ángeles Moreno de la Higuera, Aurora Viloria Jimenez, Rocío Fernández Díaz, Daniel Parra Corral, Paula Gutiérrez Sanjuan, Virginia López de la Manzanara, Diana Gimeno Álvarez, Pilar Matía, María Gemma Hernández Núñez, María Rosario Pino, Miguel Archanco Olcese, Patricia Fuente Lozoya, Laura Lopez Gonzalez, Natividad Calvo Romero, Isabel Pérez Flores, Arianne Aiffil Maneses, Ana Isabel Sánchez Fructoso

**Affiliations:** ^1^ Department of Nephrology, Hospital Clínico San Carlos, Instituto de Investigación Sanitaria San Carlos (IdISSC), Madrid, Spain, madrid.org; ^2^ Department of Palliative Care, Hospital Clínico San Carlos, Instituto de Investigación Sanitaria San Carlos (IdISSC), Madrid, Spain, madrid.org; ^3^ Nephrology Nursing Unit, Hospital Clínico San Carlos, Instituto de Investigación Sanitaria San Carlos (IdISSC), Madrid, Spain, madrid.org; ^4^ Department of Nephrology, Clinical Psychology, Hospital Clínico San Carlos, Instituto de Investigación Sanitaria San Carlos (IdISSC), Madrid, Spain, madrid.org; ^5^ Department of Psychiatry, Hospital Clínico San Carlos, Instituto de Investigación Sanitaria San Carlos (IdISSC), Instituto de Investigación Sanitaria San Carlos (IdISSC), Madrid, Spain, madrid.org; ^6^ Department of Endocrinology and Nutrition, Hospital Clínico San Carlos, Instituto de Investigación Sanitaria San Carlos (IdISSC), Madrid, Spain, madrid.org; ^7^ Clinical Dietetics Unit, Endocrinology and Nutrition, Hospital Clínico San Carlos, Instituto de Investigación Sanitaria San Carlos (IdISSC), Madrid, Spain, madrid.org; ^8^ Department of Physical Medicine and Rehabilitation, Hospital Clínico San Carlos, Instituto de Investigación Sanitaria San Carlos (IdISSC), Madrid, Spain, madrid.org; ^9^ Department of Nephrology, Social Work Unit, Hospital Clínico San Carlos, Instituto de Investigación Sanitaria San Carlos (IdISSC), Madrid, Spain, madrid.org; ^10^ Department of Microbiology and Infectious Diseases, Hospital Clínico San Carlos, Instituto de Investigación Sanitaria San Carlos (IdISSC), Madrid, Spain, madrid.org

**Keywords:** kidney transplantation, multidisciplinary care, NECPAL, quality of life, spirituality

## Abstract

**Background:**

Kidney transplant recipients with high clinical complexity often face persistent symptom burden, emotional distress, and reduced quality of life despite stable graft function. Evidence for structured multidisciplinary programs tailored to this population remains scarce.

**Methods:**

We conducted a 12‐month, single‐center implementation cohort study including 73 kidney transplant recipients classified as complex using NECPAL criteria or presenting significant emotional distress. Participants were enrolled in a coordinated, person‐centered multidisciplinary program integrating nephrology, psychology, palliative care, nutrition, rehabilitation, nursing, and social work. Outcomes were assessed at baseline and Months 1, 3, 6, 9, and 12 using validated instruments: ESAS‐r (symptom burden), DME (emotional distress), GES (spiritual well‐being), and SF‐12 (health‐related quality of life). Global adaptation was defined as improvement in ≥ 2 of 3 domains (symptom burden, emotional distress, and spirituality). Qualitative data from open‐ended responses were thematically analyzed.

**Results:**

Over follow‐up, participants showed reductions in symptom burden (ESAS‐r: 35.27 to 18.60), emotional distress (DME: 12.53 to 6.57), and increases in spiritual well‐being (GES: 17.21–20.12), with improvements in SF‐12 general health, emotional health, and social functioning. Global adaptation was achieved by 43.9% of patients, most within 3 months. Patients with ≥ 4 NECPAL criteria exhibited the most pronounced multidimensional gains. Emotional coping emerged as the strongest predictor of improvement. Qualitative analysis revealed unreported symptoms, resilience strategies, and existential reframing.

**Conclusions:**

Implementation of a structured multidisciplinary model, combined with early complexity screening, was feasible and associated with longitudinal improvements across physical, emotional, spiritual, and functional domains in complex kidney transplant recipients. These preliminary findings suggest potential benefit—particularly for those with highest baseline vulnerability—and support the need for validation in multicenter, controlled studies.

## 1. Introduction

Kidney transplantation is the treatment of choice for most patients with advanced chronic kidney disease (CKD), offering improved survival and quality of life compared with dialysis. However, a substantial proportion of recipients—particularly those with high clinical and psychosocial complexity—continue to experience persistent symptom burden, emotional distress, and impaired quality of life despite stable graft function. These challenges often arise from multimorbidity, treatment‐related complications, and the cumulative impact of functional decline, uncertainty, and existential disruption [1].

Conventional post‐transplant follow‐up primarily focuses on graft function, immunosuppression management, and surveillance for complications. While essential, this approach may overlook multidimensional needs, leaving physical, psychological, social, and spiritual aspects of well‐being insufficiently addressed [2, 3]. Previous observational studies have documented high prevalence of pain, fatigue, anxiety, depression, and spiritual distress among kidney transplant recipients, but structured longitudinal multidisciplinary programs remain scarce [4, 5].

The NECPAL tool has been validated as a method for early identification of patients with advanced chronic conditions who may benefit from supportive or palliative care [6, 7]. By integrating clinical, functional, emotional, and prognostic indicators, NECPAL enables stratification of complexity and may support timely, proactive interventions in renal transplant settings [8]. Although multidisciplinary models have shown promise in CKD populations, their structured application in kidney transplant recipients—particularly those identified as complex—has been limited [5, 9–11].

### 1.1. Objective

This study describes the implementation of a structured, person‐centered multidisciplinary care model combined with early complexity screening using the NECPAL tool. We report longitudinal changes in symptom burden, emotional distress, spiritual well‐being, and health‐related quality of life in a cohort of clinically complex kidney transplant recipients.

## 2. Methods

### 2.1. Study Design and Setting

We conducted a prospective, observational cohort study at a tertiary university hospital in Madrid, Spain, between March 2024 and April 2025. The intervention was delivered within a dedicated multidisciplinary clinic for patients with kidney transplantation identified as having complex needs.

### 2.2. Participants

Eligible participants were adult kidney transplant recipients (≥ 18 years), including recent, chronic, or complicated cases, followed in the nephrology service. Inclusion criteria were as follows:

 ≥ 1 positive NECPAL CCOMS‐ICO criterion including significant emotional distress (defined by DME score above the established threshold).
 Ability to participate in multidisciplinary evaluation.
 Signed informed consent.

Exclusion criteria included the following: cognitive impairment precluding scale completion, language barriers, unstable follow‐up, or refusal to participate.

Additionally, exclusion criteria included referrals primarily driven by transient acute events (e.g., short‐lived intercurrent illness or post‐hospitalization issues) where improvement with routine transplant follow‐up was expected and multidisciplinary follow‐up was not deemed necessary.

### 2.3. Complexity Screening (NECPAL Tool)

The NECPAL tool integrates clinical, functional, emotional, and prognostic indicators to identify patients with advanced chronic conditions who may benefit from supportive care. For each patient, the “surprise question” was combined with additional clinical indicators to determine NECPAL status and complexity level (1, 2‐3, and ≥ 4 positive criteria) [6, 7]. NECPAL criteria were assessed at baseline for eligibility and stratification and were not reassessed as a longitudinal outcome measure.

### 2.4. Intervention

Participants received a structured, person‐centered multidisciplinary intervention involving nephrology, psychology, palliative care, nutrition, rehabilitation, nursing, and social work. Key components included the following:

 Comprehensive baseline multidimensional assessment.
 Tailored interventions for symptom control, emotional support, nutritional optimization, functional rehabilitation, social guidance, and spiritual care when needed.
 Advance care planning (ACP) conversations where appropriate.

Follow‐up visits were scheduled at baseline and 1, 3, 6, 9, and 12 months, delivered in person or via teleconsultation, with flexible scheduling to ensure continuity of care. When complex needs were considered resolved, patients transitioned back to routine transplant follow‐up after a closing visit, with the option for rereferral if new needs emerged; patients with persistent complex needs continued multidisciplinary follow‐up as clinically indicated.

### 2.5. Follow‐Up Schedule and Assessments

All participants were followed for at least 12 months under the study protocol. Patient‐reported outcomes and symptom assessments (ESAS‐r, DME, GES, and SF‐12) were collected at predefined timepoints (baseline and Months 1, 3, 6, 9, and 12), regardless of whether complex needs were considered clinically resolved before Month 12.

### 2.6. Outcomes

The primary outcome was longitudinal change in multidimensional status (symptom burden, emotional distress, spiritual well‐being, and health‐related quality of life) over 12 months, overall and stratified by the NECPAL group.

Secondary outcomes included the following:

 Evolution of specific ESAS symptoms.
 Proportion achieving global adaptation (improvement in ≥ 2 of 3 domains: ESAS, DME, and GES).
 Predictors of improvement.
 Qualitative insights from open‐ended responses.

### 2.7. Assessment Tools

Assessment tools included the following:

 NECPAL tool: NECPAL CCOMS‐ICO criterion. NECPAL stratification incorporated nine validated domains: emotional distress, persistent symptoms, multimorbidity/resource use, the “surprise question,” palliative demand, disease progression, clinical decline, social vulnerability, and geriatric syndromes, with patients classified according to the total number of positive indicators [6, 7].
 ESAS‐r: Edmonton Symptom Assessment Scale‐renal version [12–15].
 DME: Emotional Distress Detection Scale [16].
 GES: Spirituality Evaluation Guide [17].
 SF‐12: Health‐related quality of life [18].

Additional baseline measures included Charlson Comorbidity Index, Clinical Frailty Scale, Barthel Index, Pfeiffer Test, Gijón Socio‐Family Risk Index, and MUST.

### 2.8. Statistical Analysis

Continuous variables were summarized as mean ± SD or median (IQR) and compared using *t*‐tests, ANOVA, Wilcoxon, or Kruskal–Wallis tests as appropriate. Categorical variables were presented as counts (%) and compared using *χ* ^2^ or Fisher’s exact tests. Longitudinal changes were assessed with Friedman or repeated‐measures ANOVA tests. Exploratory chained linear regression explored sequential relationships between domains; binary logistic regression identified predictors of global adaptation. Significance was set at *p* < 0.05. Analyses were performed with SPSS v25 and Python 3.2. Longitudinal analyses were conducted using available paired observations at each timepoint; no imputation of missing follow‐up data was performed.

### 2.9. Ethics

The study was approved by the Ethics Committee for Clinical Research of Hospital Clínico San Carlos (Protocol ID: 23/163‐E) and conducted in accordance with the Declaration of Helsinki and the General Data Protection Regulation (EU 2016/679). All participants provided written informed consent.

## 3. Results

### 3.1. Patient Characteristics

A total of 73 kidney transplant recipients with complex needs were enrolled between March 2024 and April 2025. Median age was 63 years (IQR: 49.5–73.0); 39.7% were male. At the time of analysis, 60.3% remained under active multidisciplinary follow‐up; 13.7% had transitioned back to routine transplant follow‐up after resolution of complex needs following a closing visit; 19.2% had died; and 6.8% had incomplete follow‐up due to logistical barriers. Regarding education, 60.6% had only primary or secondary studies, and 19.2% were actively employed at baseline. Social vulnerability, assessed using the Gijón Socio‐Family Risk Index, was present in 19.1% of participants. Stable family support was reported by 94.5%, and adequate financial resources by 87.7%. Spiritual beliefs were reported by 52.1%, although only a minority were practicing. Clinically, all were kidney transplant recipients (32.75% chronic, 8.2% recent, 36.9% with major comorbidities, 15.4% predialysis, and 6.8% with early graft loss). Main etiologies included interstitial nephropathy (21.9%), glomerulonephritis (16.4%), polycystic kidney disease (13.7%), and diabetic nephropathy (12.3%). Post‐transplant complications included serious infections (49.3%), neoplasms (24.7%), graft rejection (15.1%), and urological issues (15.1%) (Table 1). Many patients presented vulnerability indicators: Charlson Comorbidity Index ≥ 6.8, Clinical Frailty Scale ≥ 5 (31%), Barthel Index moderate‐to‐severe dependence (24.7%), and MUST ≥ moderate risk (29%). During follow‐up, patients received tailored interventions covering clinical and psychoemotional domains. The most frequent were ACP (77.6%) and psychological support (65.7%), followed by pharmacological treatment for depression (47.8%), anxiety (43.3%), and pain (34.3%). Nutritional counseling, social work guidance, and management of anorexia and dyspnea were also common.

**TABLE 1:** Baseline clinical and sociodemographic characteristics of the study cohort (*N* = 73).

Baseline characteristics	*N* = 73
Age, years (median, IQR)	63 (49.5–73.0)
Male gender, *n* (%)	29 (39.7)
Educational level, *n* (%)	
Primary/secondary	44 (60.6)
University	24 (33.3)
Currently employed, *n* (%)	14 (19.2)
Spiritual beliefs, *n* (%)	38 (52.1)
Christianity	16 (21.9)
Other	22 (30.1)
Strong family support, *n* (%)	45 (61.6)
Main caregiver, *n* (%)	
Partner	34 (46.6)
Children	16 (21.9)
Adequate financial resources, *n* (%)	64 (87.7)
Primary kidney disease, *n* (%)	
Immunologic	17 (23.3)
NTIC	15 (20.5)
Diabetic nephropathy	9 (12.3)
Polycystic kidney disease (PKD)	8 (11.0)
Renal Transplant stage at baseline, *n* (%)	
Renal transplant CKD predialysis	11 (15.4)
Recent transplant	6 (8.2)
Chronic transplant	24 (32,75)
Transplant with comorbidity	27 (36.9)
Early graft loss	5 (6.8)
Years since transplant (median, IQR)	6.1 (2.1–16.5)
Post‐transplant complications, *n* (%)	
Infectious	36 (49.3)
Neoplasia	18 (24.7)
Urological	11 (15.1)
Rejection	11 (15.1)
Chronic diarrhea	9 (12.3)
Institutionalized, *n* (%)	3 (4.1)

*Note:* Values are presented as absolute frequency and percentage unless otherwise indicated.

Abbreviations: IQR = interquartile range, NTIC = nontraditional immune causes, and PKD = polycystic kidney disease.

### 3.2. Primary Outcome: Overall and by NECPAL Stratification

#### 3.2.1. Longitudinal Changes in Core Patient‐Reported Outcomes (ESAS‐r, DME, GES, and SF‐12)

**FIGURE 1:**
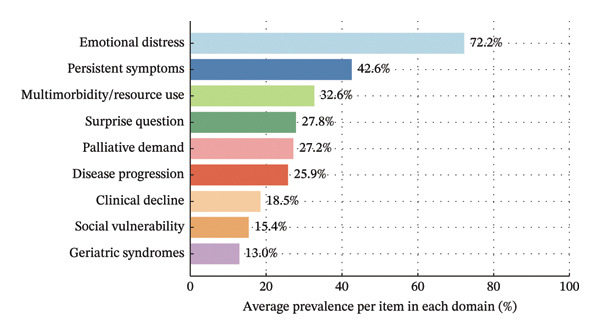
Longitudinal evolution of selected ESAS symptoms. Line chart depicting mean scores for selected physical and emotional symptoms from the Edmonton Symptom Assessment Scale (ESAS‐r) at baseline and during follow‐up. The greatest improvements were observed in anxiety, anorexia, and sadness by Month 6, with sustained effects through Month 12.

Symptom burden (ESAS‐r): Mean total score decreased from 35.27 to 18.60 at Month 12 (Δ = −16.67; *p* < 0.01), a 47.3% reduction. Most changes occurred by Month 6 and were sustained. Largest improvements were in anxiety (Δ = −3.86; *p* < 0.001), anorexia (Δ = −2.60; *p* = 0.004), sadness (Δ = −2.37; *p* < 0.05), and insomnia (Δ = −2.00; *p* = 0.002) (Figure 1).

**FIGURE 2:**
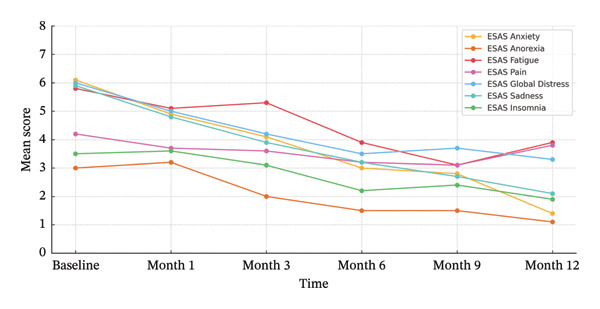
Evolution of emotional distress and related concerns (DME dimensions). Line chart showing the scaled progression of emotional distress (DME), mood, coping, and types of patient‐reported concerns from baseline to 12 months. Notable improvements were observed in total distress, coping ability, and the frequency of emotional, familial, and somatic concerns.

Emotional distress (DME): Total scores dropped from 12.53 to 6.57 by Month 6 (Δ = −5.96; *p* < 0.0001), remaining stable thereafter. Notable improvements were observed in mood (Δ = −3.14) and coping (Δ = −2.83), both *p* < 0.0001. Reductions in emotional concerns (45.4%⟶18.8%) and familial concerns (24.2%⟶10.4%) were significant (Figure 2).

**FIGURE 3:**
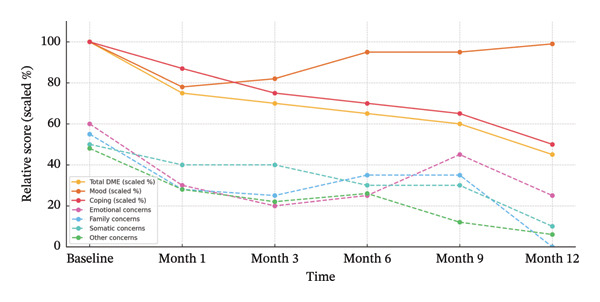
Evolution of spiritual well‐being (GES dimensions). Line chart showing the mean progression of grouped spirituality domains based on the Spirituality Evaluation Guide (GES), from baseline to 12 months. Notable improvements were observed across all existential dimensions, particularly in satisfaction, life meaning, spiritual connection, and perceived relational peace.

Spiritual well‐being (GES): It increased from 17.21 to 20.12 at Month 6 (Δ = +2.91; *p* = 0.0257), with gains in meaning (+0.62; *p* = 0.0186), hope (+0.63; *p* = 0.0143), and spiritual connection (+0.55; *p* < 0.05) (Figure 3).

**FIGURE 4:**
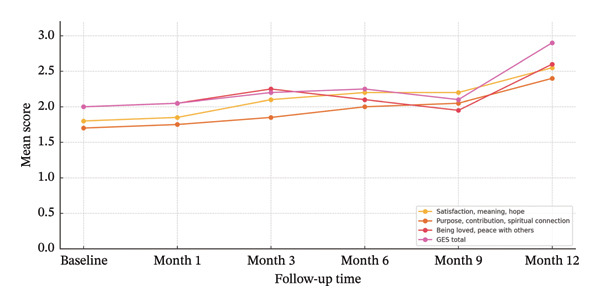
Longitudinal evolution of SF‐12 dimensions. Line chart showing the mean scores for five key dimensions of the SF‐12 (general health, physical function, emotional well‐being, pain, and social impact) from baseline to Month 12. Shaded areas represent approximate standard error. Asterisks indicate statistically significant improvements compared to baseline (*p* < 0.05).

Quality of life (SF‐12): General health improved from 2.07 to 3.00 (*p* = 0.025), emotional health from 2.13 to 2.48 (*p* = 0.0224), and social functioning from 2.72 to 5.20 (*p* < 0.001). Composite SF‐12 index rose from 1.81 to 2.22, with a shift from high‐ to moderate‐risk profiles (Figure 4).

### 3.3. NECPAL Stratification and Clinical Profiles

**FIGURE 5:**
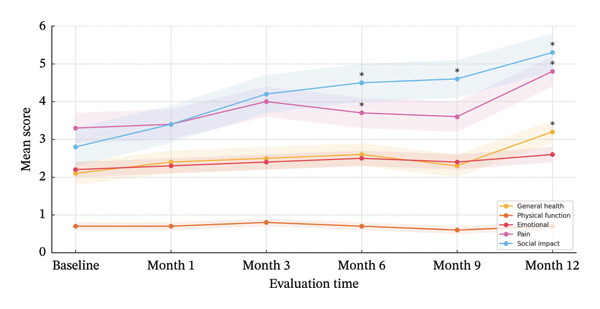
Horizontal bar chart representing the average presence (%) of each NECPAL domain among the study population (*N* = 73). Emotional distress and persistent symptoms were the most frequently reported domains, followed by multimorbidity, perceived deterioration, and social or functional vulnerability.

All patients were NECPAL‐positive. The most frequent indicators were emotional distress (74.0%), multimorbidity (≥ 3 conditions, 41.1%), repeated hospitalizations (34.2%), and poor prognosis (30.1%) (Figure 5). Patients were stratified into three subgroups according to the number of positive NECPAL indicators: 1 (low complexity; 38.4% of the cohort), 2‐3 (intermediate complexity; 31.5%), and ≥ 4 (high complexity; 30.1%). This allowed identification of distinct clinical profiles.

**FIGURE 6:**
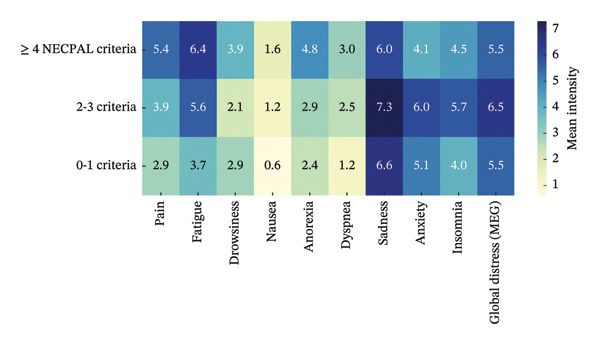
Symptom intensity by NECPAL group (ESAS baseline profiles). Heatmap displaying the mean intensity of baseline symptoms assessed by the ESAS‐r, stratified by NECPAL complexity groups (0‐1, 2‐3, and ≥ 4 criteria). Higher symptom burden was observed in patients with greater clinical complexity, particularly in pain, fatigue, sadness, and global distress.

At baseline, physical symptoms (pain, dyspnea, and fatigue) increased progressively with clinical complexity: mean intensity 2.7 in the 1‐criterion group, 3.5 in the intermediate group, and 4.4 in the ≥ 4‐criteria group (*p* = 0.008). In contrast, emotional symptoms (anxiety, sadness, and insomnia) were more pronounced in the less complex groups (mean 5.2 in the 1‐criterion group; 6.4 in the 2‐3‐criteria group vs. 4.9 in the ≥ 4‐criteria group; *p* = 0.042). This pattern suggests two distinct clinical profiles: an “emotional” profile in patients with lower disease burden and a “physical” profile in those with higher clinical complexity. Patients with ≥ 4 criteria also had significantly higher baseline symptom burden (ESAS mean: 43.1), emotional distress (DME mean: 13.35), and lower functional perception (SF‐12 mean: 1.69), compared to those with fewer criteria (Figure 6).

The intervention led to a significant reduction in total ESAS scores across all groups. The ≥ 4 criteria group improved from 43.1 to 26.4 (Δ = −16.7; *p* = 0.003), the 2‐3 group from 38.6 to 26.1 (Δ = −12.5; *p* = 0.021), and the 0‐1 group from 33.8 to 25.5 (Δ = −8.3; *p* = 0.048).

In the most complex group, clinically meaningful improvements were observed in pain (5.4–1.0; *p* < 0.01), anxiety (4.95–2.14; *p* < 0.01), sadness (4.10–2.14; *p* = 0.0187), and general malaise (5.45–3.43; *p* = 0.0486). Sustained improvements in emotional symptoms were also noted across all groups.

Patients with ≥ 4 criteria had also experienced the most marked and statistically significant improvements across all other domains by Month 12, including emotional distress (Δ = −6.47; *p* < 0.001) and spiritual well‐being (Δ = +4.35; *p* = 0.034). The intermediate group (2‐3 criteria) showed consistent multidimensional benefit, with significant gains in symptoms and emotional and functional outcomes. Patients with 1 criterion exhibited lower initial burden and improved primarily in emotional distress (Δ = −6.86; *p* = 0.015), with more modest gains in other dimensions.

**FIGURE 7:**
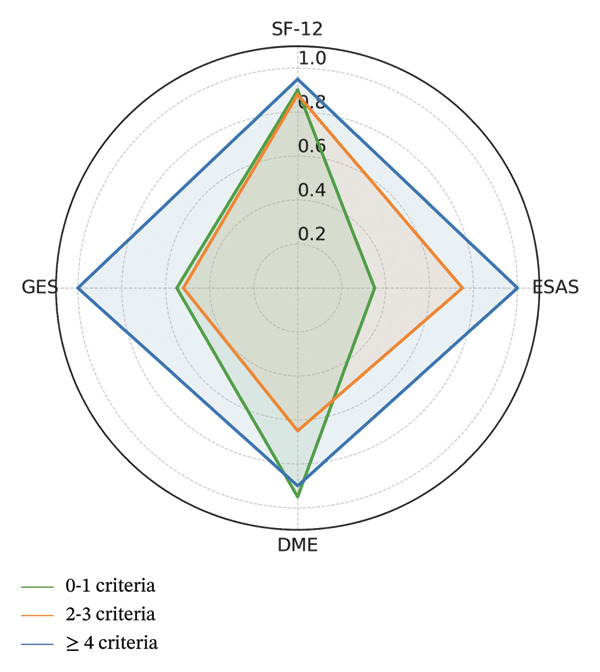
Normalized clinical improvement profile by NECPAL group (baseline to Month 3/6). Radar plot showing the relative improvement (0%–100% scale) across four key domains (symptoms [ESAS], emotional distress [DME], spiritual well‐being [GES], and functional health [SF‐12]) by NECPAL subgroup. Patients with ≥ 4 criteria showed the greatest multidimensional gains, particularly in symptom relief and spirituality.

Overall, the data confirm that while all complexity groups benefited from the intervention, the most substantial and clinically relevant gains occurred in those with the highest baseline needs (Figure 7).

### 3.4. Global Adaptation

**FIGURE 8:**
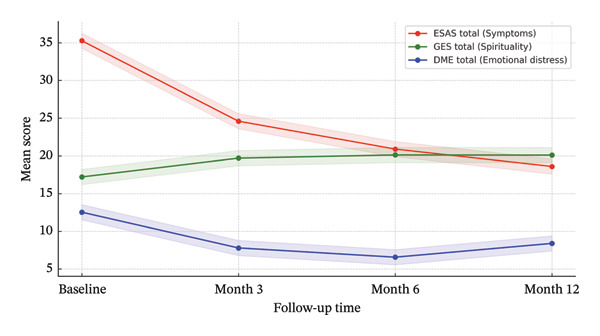
Longitudinal evolution of global outcome scores (ESAS, DME, and GES). Line chart illustrating the evolution of global symptom burden (ESAS), emotional distress (DME), and spiritual well‐being (GES) from baseline to Month 12 (Global Adaptation Index). Statistically significant improvements were observed in all three domains, particularly during the first 6 months of follow‐up.

43.9% met the criteria for global adaptation. Most (77.8%) achieved this by Month 3. These patients had higher baseline symptom scores (ESAS: 128.8 vs. 101.7; *p* = 0.004), emotional distress (DME: 13.9 vs. 11.4; *p* = 0.018), and spirituality (GES: 14.8 vs. 11.2; *p* = 0.015). Longitudinal trajectories of global symptom burden (ESAS), emotional distress (DME), and spiritual well‐being (GES) are shown in Figure 8.

A binary logistic regression model was performed to identify independent predictors of global adaptation. Age was significantly associated with likelihood of adaptation (*p* = 0.002), while higher baseline scores in ESAS (*p* = 0.004) and DME (*p* = 0.021) also increased the probability of achieving multidimensional improvement. Baseline spirituality (GES) emerged as a positive modulator (*p* = 0.015), suggesting that emotional and existential reserves may play a protective role. No significant associations were found with sex, socioeconomic status, family support, NECPAL group classification, or renal disease status. No statistically significant differences were identified between groups with respect to tacrolimus trough levels, renal function parameters (including serum creatinine and estimated glomerular filtration rate), or other routine laboratory values (Table 2).

**TABLE 2:** Univariate binary logistic regression predicting global adaptation.

Variable	Beta coefficient (log‐odds)	*p* value
Age (years)	−0.06342	0.007
Male gender	−0.37788	0.541
Renal transplant stage at baseline		
Renal transplant CKD predialysis	−1.09861	0.472
Early graft loss	−22.8219	0.999
Transplant with severe comorbidity	−1.8718	0.136
Chronic transplant	−1.09861	0.394
Recent transplant	−24.5475	0.999
Family support	−0.1699	0.783
Financial resources	−1.02962	0.425
Spirituality	0.093526	0.885
NECPAL 2–3 criteria	−0.04879	0.951
NECPAL ≥ 4 criteria	−0.18492	0.790
Baseline ESAS total	0.012058	0.068
Baseline DME coping	0.170572	0.021

*Note:* The table shows beta coefficients (log‐odds) and *p* values from univariate binary logistic regression. Only age and baseline emotional distress (DME total) were significantly associated with the probability of achieving global adaptation. Clinical stage, NECPAL criteria, and social or demographic variables were not significant predictors.

Despite these patterns, NECPAL classification was not a statistically significant predictor of global adaptation in multivariate analysis, suggesting that while NECPAL criteria [11] help stratify initial risk and clinical complexity, the intervention model is beneficial across all profiles [19], including those with the highest clinical complexity.

### 3.5. Predictors of Improvement

**FIGURE 9:**
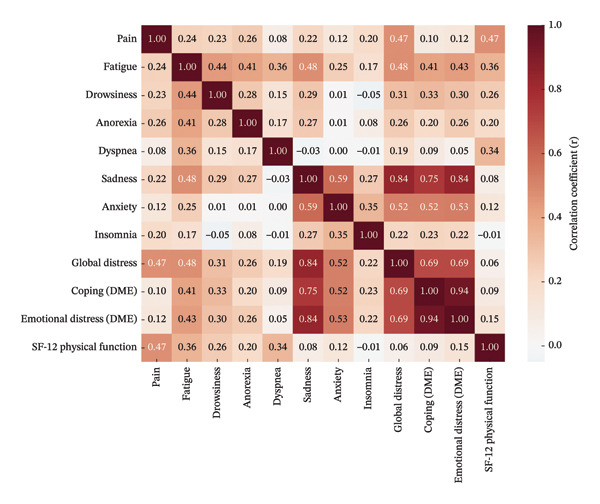
Correlation matrix among symptom, emotional, and functional domains (baseline). Heatmap displaying Pearson correlation coefficients between ESAS symptoms, DME components (coping and emotional distress), and SF‐12 physical functioning at baseline. Strong associations were observed between sadness, anxiety, global distress (MEG), and emotional coping, reflecting the interconnected nature of physical and emotional suffering in this population.

Baseline correlations among symptom, emotional, and functional domains are presented in Figure 9. In an exploratory chained regression analysis, reduction in physical symptoms (e.g., pain and insomnia) predicted improvement in emotional domains (anxiety *β* = 0.59; sadness *β* = 0.50), which in turn predicted enhanced coping (*β* = 0.71). Emotional coping emerged as the strongest predictor of global distress reduction (*β* = 1.58; *R* ^2^ = 0.806; *p* < 0.001).

### 3.6. Qualitative Data and Open‐Ended Responses

In addition to structured quantitative assessments, open‐ended responses were systematically collected within the ESAS‐r, DME, and GES tools. These free‐text fields captured subjective narratives of suffering and resilience that complemented standardized scores. Patients spontaneously reported symptoms such as tremors, cramps, and gastrointestinal discomfort not included in the ESAS scale, underscoring the value of open expression.

In the emotional and existential domains, patients described feelings of fear, hopelessness, guilt, and gratitude. Responses revealed deep concerns about dependency, family burden, loss of autonomy, and disrupted life trajectories. Several patients used these open spaces to articulate spiritual experiences, symbolic interpretations of illness, and metaphors of transformation.

**FIGURE 10:**
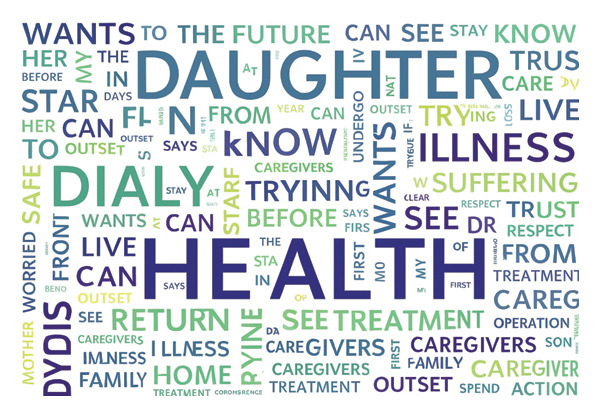
Word cloud—emotional distress and existential themes.

To synthesize the content, word clouds were generated using natural language processing tools to visualize the most frequent and emotionally charged terms. Terms such as “pain,” “daughter,” “fear,” “God,” “health,” and “dialysis” emerged as dominant themes. This qualitative insight enriched the understanding of multidimensional distress and reinforced the need for personalized, narrative‐sensitive interventions within the multidisciplinary model (Figure 10).

These findings illustrate that structured tools alone may not fully capture the complexity of suffering in transplant patients, and that open dialog—through both verbal and symbolic language—serves as a therapeutic bridge to meaning‐making, adaptation, and care alignment.

In summary, early complexity screening with NECPAL revealed distinct “emotional” and “physical” symptom patterns in kidney transplant recipients with complex needs. A structured, multidisciplinary intervention was associated with sustained multidimensional benefits, with coping emerging as a key mediator in a sequential pathway from physical symptom relief to emotional recovery. Qualitative insights added depth, uncovering unmet needs and resilience narratives, underscoring the value of integrating complexity screening, targeted symptom management, and narrative‐sensitive care in transplant follow‐up.

## 4. Discussion

This study suggests that an anticipatory, person‐centered multidisciplinary model is feasible and was associated with clinically relevant improvements in multidimensional outcomes among kidney transplant recipients with complex clinical and psychosocial profiles. Improvements were observed across physical symptoms, emotional distress, spirituality, and perceived quality of life, with sustained benefits throughout the observed 12‐month follow‐up.

Our results show elevated levels of anxiety, sadness, pain, and insomnia among transplant recipients with complex profiles, particularly at baseline. These findings are consistent with those reported by other authors, who also observed persistent emotional distress and reduced quality of life in kidney transplant recipients, even after successful transplantation [20, 21]. Patients with the highest baseline complexity showed the most significant and sustained improvements, particularly in pain, sadness, anxiety, and spiritual meaning. This reinforces the idea that complexity does not preclude adaptation when supported by structured multidisciplinary care. Similar benefits have been reported in CKD populations through models like KidneyPal [19]; our findings extend this evidence to transplant recipients, who also could remain clinically vulnerable despite stable graft function [22].

The stratified analysis by NECPAL criteria allowed for the identification of distinct clinical profiles and highlighted that the intervention was beneficial across all subgroups. Patients with intermediate NECPAL scores exhibited balanced, multidimensional responses, while those with fewer criteria reported gains mostly in emotional domains. Notably, NECPAL classification was not an independent predictor of global adaptation, reinforcing that the intervention model is beneficial across all profiles, including those with the highest clinical complexity. This supports the growing evidence that NECPAL is a useful tool not only for early identification of patients with complex needs but also for tailoring interventions in populations with advanced renal disease, as proposed by Valenti et al. [23].

The Global Adaptation Index emerged as a meaningful outcome measure that integrates changes in physical, emotional, and spiritual dimensions. Its early onset in most patients highlights the value of proactive care. While previous studies report persistent symptom burden after transplantation such as Tucker et al. [24] or Villeneuve et al. [25], our findings suggest that structured multidisciplinary follow‐up can facilitate early and sustained multidimensional improvement.

Predictors of adaptation—such as age, symptom burden, and baseline spirituality—highlight the value of identifying patients with latent adaptive capacity. Prior studies have linked spiritual well‐being to greater resilience [26] and emphasized the importance of narrative and qualitative approaches in understanding patient needs and lived experiences post‐transplant [27–30]. These approaches underscore the importance of acknowledging personal meaning, vulnerability, and identity reconstruction as key dimensions of post‐transplant adaptation.

The chained regression analysis provided a conceptual model for understanding the dynamic sequence of improvement: physical symptom control facilitated emotional regulation, which enhanced coping and, potentially, existential integration. In this context, the identification of symptom clusters (pain–sleep disturbances–fatigue and depression–anxiety–anorexia) has emerged as a useful framework for guiding more targeted and effective interventions [31]. The central role of emotional coping in global improvement reinforces its relevance as a clinical target. These findings align with growing evidence highlighting the need to address psychosocial distress and emotional burden across the transplant continuum and to integrate psychiatric and psychosocial care into multidisciplinary models to promote resilience and meaning‐making [32–34].

The integration of qualitative data enriched our findings by capturing hidden symptoms and the existential dimension of illness, often missed by standardized tools. Patients’ narratives revealed metaphors of suffering and recovery, existential isolation, and the search for meaning. These results align with prior qualitative work in transplantation and support the use of narrative‐sensitive approaches alongside patient‐reported outcome measures tailored to transplant populations [35, 36].

In summary, our findings advocate for the incorporation of anticipatory, interdisciplinary care models in routine transplant follow‐up. By addressing not only physical symptoms, these models offer a more ethical, effective, and humanizing approach to complex care [37–41].

This was a single‐center study in Spain, so transferability to other healthcare systems and sociocultural settings may be limited. However, social and sociocultural determinants were explicitly addressed within the model: social vulnerability was systematically assessed (e.g., Gijón Index) and social work was embedded in the pathway, alongside routine exploration of patients’ spiritual beliefs and value frameworks. Multicenter validation in diverse settings is warranted.

## 5. Limitations

This observational, single‐center implementation cohort has several limitations. First, the absence of a concurrent control group precludes causal inference and limits our ability to disentangle the effects of specific multidisciplinary components from nonspecific factors such as attention, clinical contact, regression to the mean, or natural fluctuations in symptom trajectories.

Second, follow‐up completeness varied in this real‐world cohort. Although all participants were scheduled to complete PROMs at baseline and Months 1, 3, 6, 9, and 12 irrespective of clinical transition back to routine follow‐up, missing data at later timepoints resulted in varying sample sizes across assessments; analyses were therefore performed using available paired observations without imputation.

Third, NECPAL criteria were used at baseline for screening and stratification and were not reassessed longitudinally, preventing evaluation of changes in complexity status over time.

Fourth, while we provide an exploratory health‐economic evaluation (Supporting Appendix 7)—including direct hospital savings, avoided “shadow cost,” and a QALY‐based valuation—this is not a formal incremental cost‐effectiveness analysis with an ICER versus a comparator group and should be interpreted as preliminary and hypothesis‐generating.

Fifth, the relatively small sample size—especially for stratified and multivariable analyses—may have limited statistical power to detect subtle associations. Finally, some domains such as spirituality or coping were measured using validated but inherently subjective tools and may be influenced by cultural or personal interpretation. As the study was conducted at a single tertiary center in Spain, generalizability to other healthcare systems and sociocultural contexts may be limited, although social vulnerability (Gijón Index) and spiritual/value frameworks were explicitly assessed and addressed within the care model.

## 6. Conclusions

In this cohort of kidney transplant recipients with complex needs, early complexity screening with the NECPAL tool revealed two distinct baseline symptom profiles: an “emotional” pattern, with higher anxiety, sadness, and insomnia in less complex patients, and a “physical” pattern, with greater pain, fatigue, and dyspnea in those with higher complexity.

The structured, person‐centered multidisciplinary intervention achieved significant and sustained improvements across symptom burden, emotional distress, spiritual well‐being, and quality of life, with the greatest benefits in the high‐complexity group (≥ 4 NECPAL criteria). Coping capacity emerged as the strongest independent predictor of global improvement, forming the final link in a “domino effect” whereby relief of physical symptoms facilitated emotional recovery, which in turn enhanced coping.

Qualitative analysis enriched these findings, revealing unreported symptoms, existential concerns, and symbolic narratives of resilience that are not captured by standardized scales. This narrative‐sensitive approach provided a deeper understanding of patient adaptation and helped to align care with individual priorities and meaning‐making processes.

These results support the integration of validated complexity screening into routine transplant follow‐up, coupled with anticipatory, holistic care models. Recognizing distinct symptom patterns, reinforcing coping strategies, and incorporating qualitative, patient‐centered insights may amplify the impact of multidisciplinary interventions, fostering resilience, engagement, and long‐term well‐being in this vulnerable population.

## Funding

This project was supported by Fundación MAPFRE (Ignacio H. de Larramendi Research Grant, 2024‐2025), Fundación Chiesi (Call for Multidisciplinary Innovation in Transplantation, 2023), Comunidad de Madrid (INVESTIGO Program, Ref. INV/2022/NEFRO‐08), and Instituto de Investigación Sanitaria San Carlos (IdISSC) (Research Support Award 2024‐2025).

## Disclosure

All interventions were delivered free of charge to patients as part of the standard comprehensive care model of the Multidisciplinary Renal Care Unit at Hospital Clínico San Carlos. The funders had no role in study design, data collection, analysis, interpretation, or manuscript preparation.

## Conflicts of Interest

The authors declare no conflicts of interest.

## Supporting Information

Additional supporting information can be found online in the Supporting Information section.

## Supporting information


**Supporting Information** Appendix 1. Exploratory economic evaluation estimate.

## Data Availability

The underlying data contain sensitive patient information and cannot be shared publicly due to privacy and ethical restrictions; deidentified data may be made available upon reasonable request from the corresponding author, subject to institutional/ethics approval as applicable.
