# Supplementary material for: Structured Multidisciplinary Care for Clinically Complex Kidney Transplant Recipients: A 12‐Month Single‐Center Cohort on Multidimensional Outcomes
Source: J Transplant. 2026 Jun 17;2026:4688022. doi: 10.1155/joot/4688022 (PMC13276289; doi:10.1155/joot/4688022)
Supplement: Supplementary file 1 — Supporting Information Appendix 1. Exploratory economic evaluation estimate. [file JOOT-2026-4688022-s001.docx]

**Appendix 1. Exploratory Economic Evaluation Estimate**

This supplementary appendix translates and reorganizes the original Spanish annex into English. It presents the economic assumptions, formulas, tabulated results, graphical summaries, and an exploratory projection of the clinic’s economic impact after 24 months of activity.

# 1. Clinical and experiential evidence supporting the assumptions

The economic assumptions are supported by the observed clinical and experiential outcomes of the multidisciplinary clinic:

- Longitudinal improvements in symptom burden, emotional distress, spiritual well-being, and health-related quality of life (ESAS, DME, GES, and SF-12).
- At end of life, a marked reduction in suffering and futile interventions, with 0% of deaths occurring in the ICU/emergency department among patients with complete palliative care/advance care planning pathways.
- Strong patient-reported experience, with high perceived improvement in anxiety, mood, self-confidence, trust in the care team, and pain management.
- External funding of psychology support and of the broader project, with no direct hospital cost for these components.

# 2. Assumptions and unit costs (editable base-case scenario)

**Table 1. Unit costs and core assumptions**

| Parameter | Base-case value / description |
| --- | --- |
| Emergency department visit | €185 |
| First specialist consultation | €132 |
| Follow-up specialist consultation | €79 |
| High-resolution multidisciplinary visit | €212 |
| ICU day | €2,301/day |
| Avoided emergency visits | 0.5 per patient-year |
| Avoided specialist consultations | 0.5 per month (mix: 20% first visits, 80% follow-up visits) |
| Avoided end-of-life ICU episodes | 0.205 episodes per deceased patient; 2 ICU days per episode (base case) |
| Reduction in deaths in emergency department | Included in the EOL component |
| Multidisciplinary clinic visit plan | 6 timepoints over 12 months: baseline, month 1, 3, 6, 9, and 12 |

# 3. Formula summary

**Table 2. Economic formulas used**

| Outcome | Formula / definition |
| --- | --- |
| Avoided ED savings (€/100 patient-years) | (ED visits avoided/patient-year) × 100 × ED unit cost |
| Avoided specialist consultation savings (€/100 patient-years) | [(consultations avoided/month) × 12] × 100 × (0.2 × €132 + 0.8 × €79) |
| Avoided EOL acute-care savings (€/100 patient-years) | [0.205 × deaths/100 patient-years × ICU days/episode × ICU unit cost] + [0.128 × deaths/100 patient-years × ED unit cost] |
| Avoided shadow cost of the multidisciplinary clinic | 6 × €212 × patient-years (high-resolution activity delivered without additional staffing cost) |
| Social value from quality-of-life gains | (Δutility × patient-years) × willingness-to-pay threshold (€25,000/QALY in the intermediate scenario) |

# 4. Economic results for the study cohort

The study cohort included 73 patients and 37.1 patient-years of follow-up.

**Table 3. Main economic results for the study cohort (N=73; 37.1 patient-years)**

| Metric | Value |
| --- | --- |
| Direct hospital savings (base case) | €36,916 |
| Sensitivity range for direct hospital savings | €16,286 to €66,897 |
| Net hospital ROI (psychologist externally funded) | €36,916 |
| Counterfactual net ROI if psychology were an internal 0.5 FTE cost | €13,028 |
| Avoided shadow cost (high-resolution clinic activity) | €47,191 |
| Expanded economic benefit (direct savings + shadow cost) | €84,107 |
| Social value from QALYs (intermediate scenario) | €27,825 |
| Total impact + social value | €111,932 |

**Table 4. Direct hospital savings breakdown**

| Component | €/100 patient-years | Study cohort (€) | Notes |
| --- | --- | --- | --- |
| Avoided ED visits | €9,250 | €3,432 | Base-case assumption: 0.5 avoided ED visits/patient-year |
| Avoided specialist consultations | €53,760 | €19,945 | Mix: 20% first visits / 80% follow-up visits |
| Avoided end-of-life acute care | €36,494 | €13,539 | ICU + emergency department component |
| Total direct savings | €99,504 | €36,916 | Rounded total |

**Table 5. Estimated avoided specialist consultation costs by specialty (€/100 patient-years)**

| Specialty | €/100 patient-years |
| --- | --- |
| Psychiatry | €16,128 |
| Gastroenterology | €10,752 |
| Rheumatology/Pain | €10,752 |
| Neurology | €8,602 |
| Geriatrics | €7,526 |


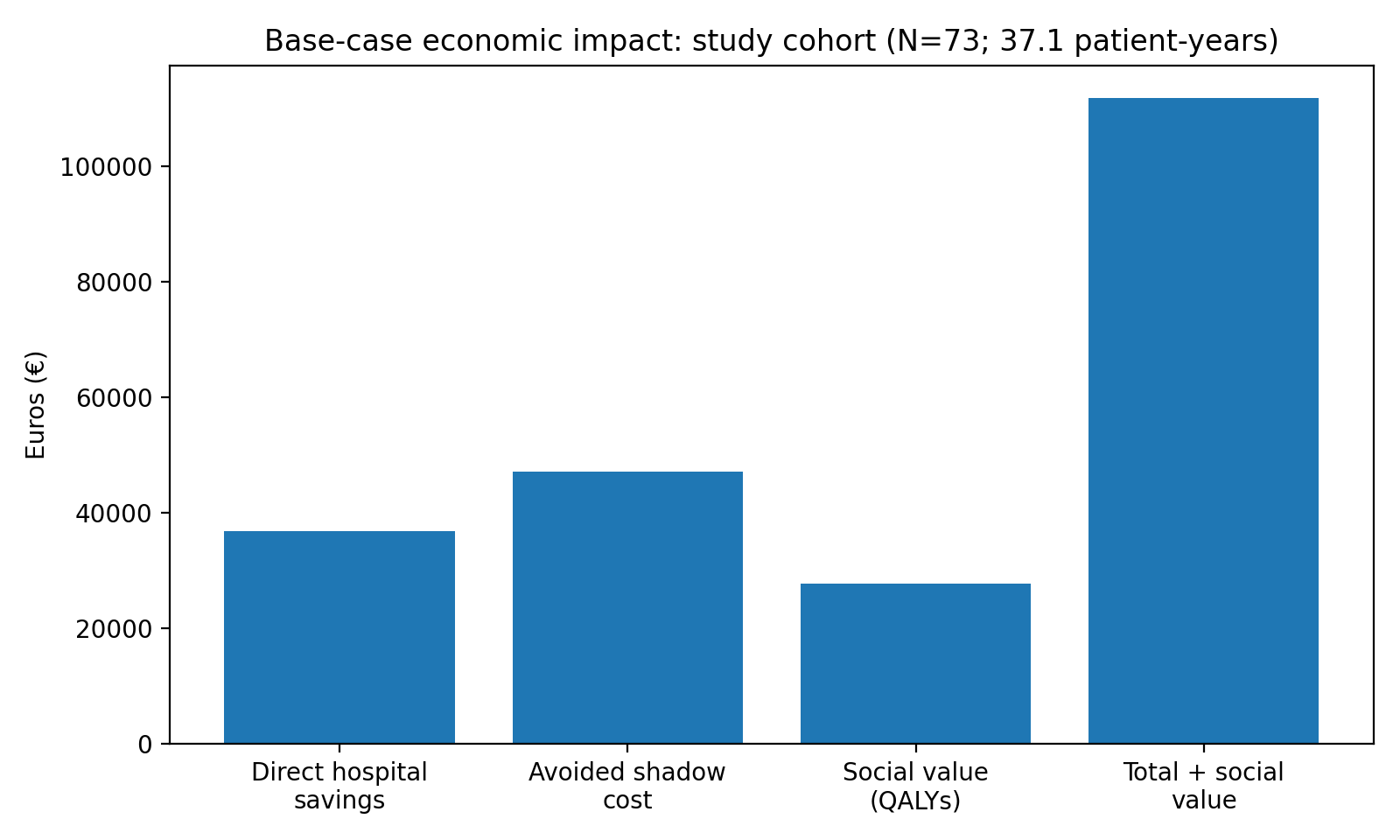


Figure 1. Base-case economic impact in the study cohort.


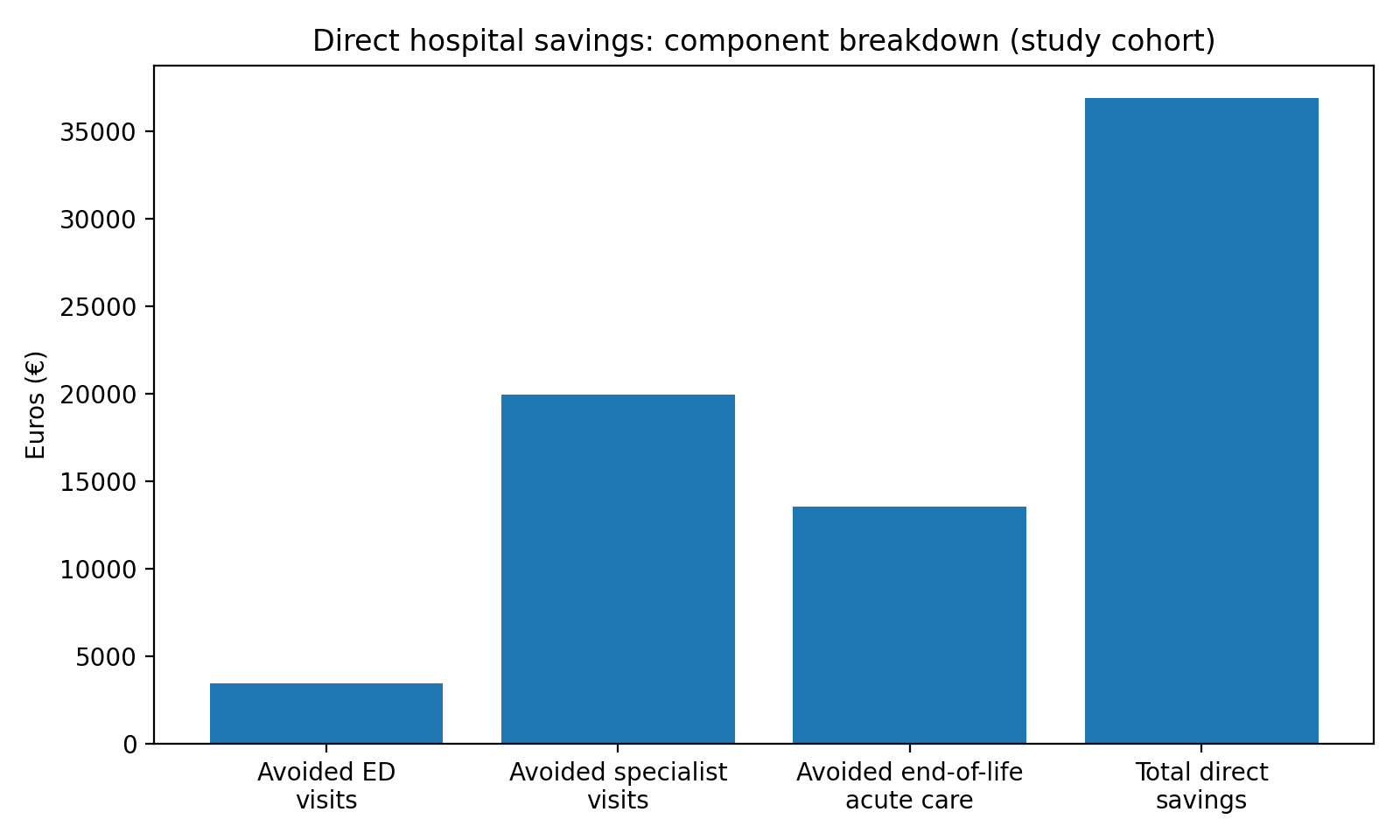


Figure 2. Direct hospital savings: component breakdown in the study cohort.

# 5. Sensitivity analysis

Sensitivity analyses varied the key assumptions as follows: avoided emergency visits 0.2–1.0 per patient-year; avoided specialist consultations 0.2–1.0 per month; and ICU length of stay 1–3 days per avoided end-of-life episode.

**Table 6. Sensitivity analysis summary**

| Parameter | Range |
| --- | --- |
| Avoided emergency visits | 0.2–1.0 per patient-year |
| Avoided specialist consultations | 0.2–1.0 per month |
| ICU days per avoided EOL episode | 1–3 days |
| Resulting direct hospital savings (study cohort) | €16,286 to €66,897 |

# 6. Exploratory projection after 24 months of clinic activity

To estimate the economic impact after two years of clinic activity, we performed an exploratory linear projection using the rescaled exposure reported in the original annex. The original rescaling for the period March 2024 to November 2025 considered 123 patients with a median follow-up of 13.8 months (approximately 141.5 patient-years). Assuming similar case mix, care intensity, and rates per 100 patient-years, we projected the exposure to 24 months (≈ 161.7 patient-years). These figures should be interpreted as an order-of-magnitude estimate rather than a formal economic evaluation.

**Table 7. Exploratory economic projection after 24 months of clinic activity**

| Metric | Estimated value | Comment |
| --- | --- | --- |
| Projected patient-years | 161.7 | Based on 141.5 patient-years over 21 months, linearly projected to 24 months |
| Direct hospital savings | €160,912 | Sensitivity range: €70,989 to €291,596 |
| Avoided shadow cost | €205,700 | High-resolution clinic activity delivered without additional staffing cost |
| Expanded economic benefit | €366,612 | Direct savings + avoided shadow cost |
| Social value from QALYs | €121,286 | Intermediate scenario, €25,000/QALY |
| Total impact + social value | €487,898 | Exploratory total |

**Table 8. Direct savings component breakdown for the 24-month projection**

| Component | Estimated value | Method |
| --- | --- | --- |
| Avoided ED visits | €14,959 | Base-case €/100 patient-years × projected patient-years |
| Avoided specialist consultations | €86,938 | Base-case €/100 patient-years × projected patient-years |
| Avoided end-of-life acute care | €59,016 | Base-case €/100 patient-years × projected patient-years |
| Total direct savings | €160,912 | Rounded total |


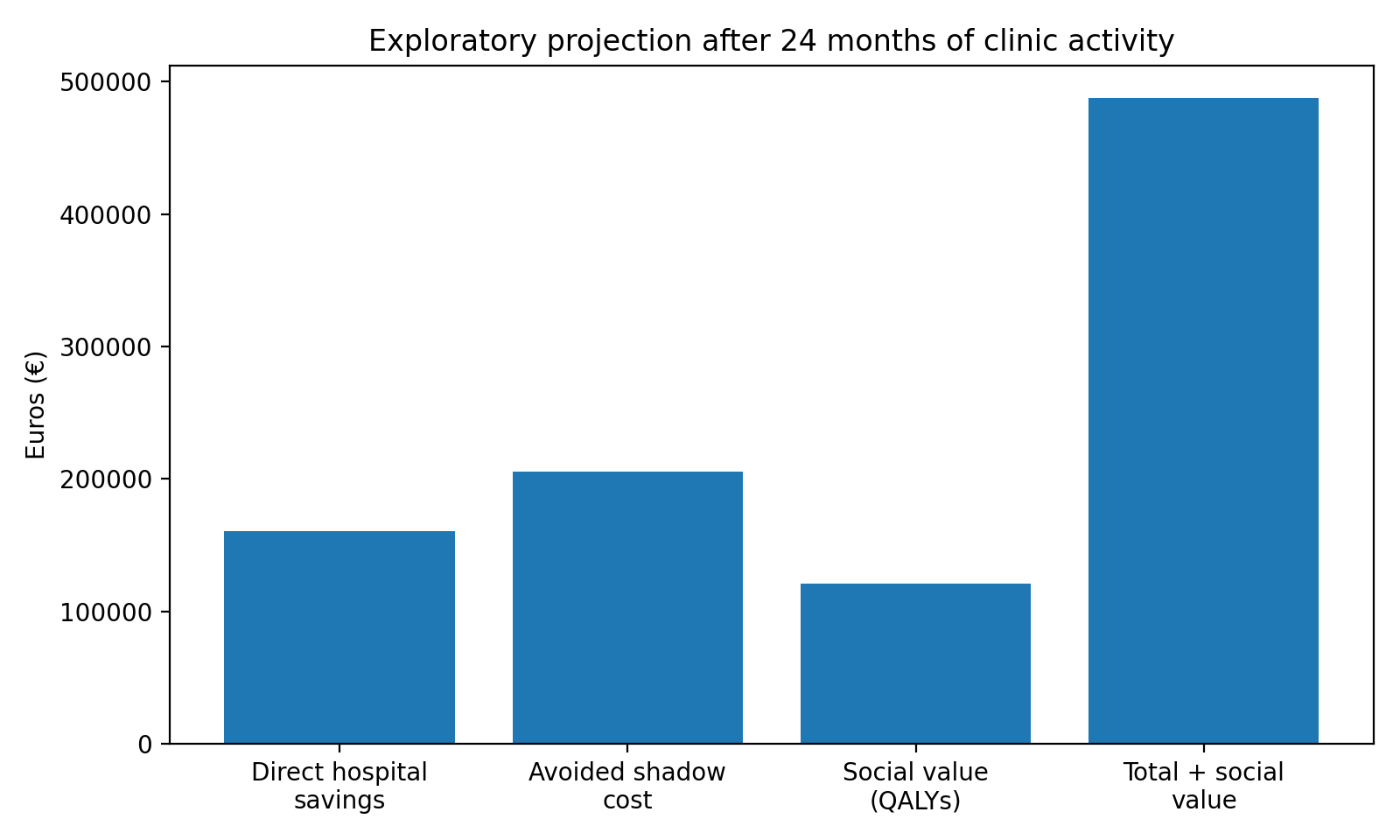


Figure 3. Exploratory projection of economic impact after 24 months of clinic activity.

# 7. Limitations and refinement needs

- Avoided readmissions and avoided diagnostic tests were not incorporated and could increase the estimated benefit if data become available.
- Caregiver impact and avoided professional coordination burden were not included.
- The 24-month projection is exploratory and assumes stability of case mix, intensity, and per-100 patient-year rates.

# 8. Summary

In the base hospital-cost scenario (psychology externally funded; multidisciplinary clinic without additional staffing cost), the study cohort generated €36,916 in direct hospital savings. When the avoided shadow cost of high-resolution activity is added, the expanded economic benefit rises to €84,107. Including the intermediate QALY-based social value estimate, the total impact reaches €111,932. Under the exploratory 24-month projection, the total impact plus social value would be approximately €487,898.
